# Altered Functional Connectivity of Salience Network in Mood Disorders

**DOI:** 10.1192/j.eurpsy.2023.1273

**Published:** 2023-07-19

**Authors:** A. Todeva-Radneva, R. Paunova, D. Stoyanov, T. Zdravkova, S. Kandilarova

**Affiliations:** Psychiatry and Medical Psychology and Research Institute, Medical University of Plovdiv, Plovdiv, Bulgaria

## Abstract

**Introduction:**

Despite the significant scientific progress in the study of the mechanisms underlying mental disorders, stable biomarkers facilitating their diagnosis and differential diagnosis are lacking. Therefore, we attempted to explore possible functional connectivity (FC) differences across some of the more prevalent mental disorders, namely mood disorders.

**Objectives:**

The current study aimed at investigating the alterations of the functional connectivity of major seeds of the Salience Network (SN) (the Anterior Cingulate Cortex (ACC), and Anterior Insula (AI) in patients with unipolar (Major Depressive Disorder-MDD) or bipolar (Bipolar Disorder-BD) depression as compared to healthy controls (HC).

**Methods:**

For this study 103 adult subjects underwent resting-state Magnetic Resonance Imaging of whom 60 were patients with a depressive episode (MDD: n=35; BD: n=25), and 43 were healthy controls (HC). The individuals in both groups were matched for age and gender. Each participant has provided written informed consent (Ethics Committee: P186/22.01.2021). Seed-to-voxel analysis was performed via the CONN toolbox running on MATLAB. Random Field Theory parametric statistics was used with a cluster-level FWE correction p<0.05.

**Results:**

Both the ACC and the right AI demonstrated a statistically significant increase in the FC to the somatosensory cortex and the motor cortex in patients as opposed to HC. In addition, there was hyperconnectivity between ACC and the right Superior Frontal Gyrus, Precuneus, and the Superior Parietal Lobule bilaterally in patients as well. A reduced FC between the ACC and the hippocampus and parahippocampus was observed in depression in comparison to HC. The analysis of the left AI seed yielded no statistically significant between-group differences.

**Conclusions:**

Our results demonstrate aberrant connectivity between nodes of the SN, the Default Mode Network, and the Limbic Network which might provide clarification on the mechanisms of impaired balance between internally and externally oriented attention, affective and cognitive control in depression. In addition, the alterations of the FC between SN and the somatosensory and motor cortices may be suggested as a possible explanation of the disturbances in the psychomotor activity in mood disorders.

**Disclosure of Interest:**

A. Todeva-Radneva Grant / Research support from: This work has been funded by the Bulgarian National Program “European Scientific Networks” - Project DIP Neuroscience, R. Paunova Grant / Research support from: This work has been funded by the Bulgarian National Program “European Scientific Networks” - Project DIP Neuroscience, D. Stoyanov Grant / Research support from: This work has been funded by the Bulgarian National Program “European Scientific Networks” - Project DIP Neuroscience, T. Zdravkova Grant / Research support from: This work has been funded by the Bulgarian National Program “European Scientific Networks” - Project DIP Neuroscience, S. Kandilarova Grant / Research support from: This work has been funded by the Bulgarian National Program “European Scientific Networks” - Project DIP Neuroscience

